# Revisiting the potential of regulated cell death in glioma treatment: a focus on autophagy-dependent cell death, anoikis, ferroptosis, cuproptosis, pyroptosis, immunogenic cell death, and the crosstalk between them

**DOI:** 10.3389/fonc.2024.1397863

**Published:** 2024-08-09

**Authors:** Maowen Luo, Xingzhao Luan, Chaoge Yang, Xiaofan Chen, Suxin Yuan, Youlin Cao, Jing Zhang, Jiaying Xie, Qinglian Luo, Ligang Chen, Shenjie Li, Wei Xiang, Jie Zhou

**Affiliations:** ^1^ Department of Neurosurgery, the Affiliated Hospital, Southwest Medical University, Luzhou, Sichuan, China; ^2^ School of Clinical Medicine, Southwest Medical University, Luzhou, Sichuan, China; ^3^ Department of Neurosurgery, the Affiliated Hospital of Panzhihua University, Panzhihua, Sichuan, China; ^4^ School of Clinical Medicine, the Affiliated Hospital of Panzhihua University, Panzhihua, Sichuan, China; ^5^ School of Clinical Medicine, Sichuan Clinical Research Center for Neurosurgery, Luzhou, Sichuan, China

**Keywords:** glioma, autophagy-dependent cell death, anoikis, ferroptosis, cuproptosis, pyroptosis, immunogenic cell death, regulated cell death

## Abstract

Gliomas are primary tumors that originate in the central nervous system. The conventional treatment options for gliomas typically encompass surgical resection and temozolomide (TMZ) chemotherapy. However, despite aggressive interventions, the median survival for glioma patients is merely about 14.6 months. Consequently, there is an urgent necessity to explore innovative therapeutic strategies for treating glioma. The foundational study of regulated cell death (RCD) can be traced back to Karl Vogt’s seminal observations of cellular demise in toads, which were documented in 1842. In the past decade, the Nomenclature Committee on Cell Death (NCCD) has systematically classified and delineated various forms and mechanisms of cell death, synthesizing morphological, biochemical, and functional characteristics. Cell death primarily manifests in two forms: accidental cell death (ACD), which is caused by external factors such as physical, chemical, or mechanical disruptions; and RCD, a gene-directed intrinsic process that coordinates an orderly cellular demise in response to both physiological and pathological cues. Advancements in our understanding of RCD have shed light on the manipulation of cell death modulation - either through induction or suppression - as a potentially groundbreaking approach in oncology, holding significant promise. However, obstacles persist at the interface of research and clinical application, with significant impediments encountered in translating to therapeutic modalities. It is increasingly apparent that an integrative examination of the molecular underpinnings of cell death is imperative for advancing the field, particularly within the framework of inter-pathway functional synergy. In this review, we provide an overview of various forms of RCD, including autophagy-dependent cell death, anoikis, ferroptosis, cuproptosis, pyroptosis and immunogenic cell death. We summarize the latest advancements in understanding the molecular mechanisms that regulate RCD in glioma and explore the interconnections between different cell death processes. By comprehending these connections and developing targeted strategies, we have the potential to enhance glioma therapy through manipulation of RCD.

## 1 Introduction

Glioma is a highly heterogeneous, aggressive primary brain tumor. The current standard of care for patients diagnosed with glioma includes performing a maximum safe extent resection of the tumor, undergoing radiotherapy over a 6-week period, and receiving concomitant systemic therapy with the alkylating agent TMZ (1). In addition to this, treatments such as immunotherapy, targeted precision therapy, electric field therapy, and supportive care have resulted in some improvement in the survival duration of glioma patients. However, these patients typically experience a poor prognosis and low quality of life, with only a 4-5% 5-year survival rate (2). Furthermore, cognitive and focal neurological deficits can significantly impact long-term survivors of brain tumors, regardless of tumor histology and grading (3).

The genesis of the scientific inquiry into RCD is often attributed to Karl Vogt’s discernment of cellular demise in toads in 1842. A significant leap in the conceptual framework occurred in 1972 when Kerr, Wyllie, and Currie introduced the term “apoptosis” to the research lexicon, propelling the study of RCD to the scientific forefront (4). The discovery by Sulston and Horvitz in 1976, utilizing the model organism C. elegans, was instrumental in unearthing the genetic underpinnings of apoptosis and revealing a predetermined fate for approximately 13% of somatic cells during embryogenesis (5). Over the past decade, NCCD has meticulously curated a taxonomy of cell death modalities, based on distinguishing cellular morphology, biochemistry, and functional outcomes. The dichotomy between cell death pathways lies within two realms: accidental pathways triggered by external factors such as mechanical, chemical, and physical stressors that lead to cellular demise when they surpass the cell’s compensatory capacities; and RCD, which represents an inherent and orchestrated cessation of cellular function mediated precisely by genetic regulation to maintain internal homeostasis. This gene-directed cessation in a physiological context is also known as programmed cell death (PCD) (6). Diverging from the unregulated nature of accidental cell death (ACD), RCD involves a meticulous network of signaling cascades and molecular machinery. The exploration of RCD has revealed numerous novel mechanisms with profound connections to human pathologies, expanding beyond apoptosis to encompass diverse processes such as autophagy-dependent cell death, ferroptosis, lysosome-dependent cell death, mitochondrial permeability transition (MPT)-driven necrosis, necroptosis, pyroptosis, anoikis, NETosis, among others (7). Each mechanism reflects a unique aspect of the cell’s ability to maintain physiological integrity and their ongoing elucidation holds significant promise in enlightening the pathogenesis of various diseases and refining therapeutic strategies.

RCD, a significant pathway in the cell death process, exhibits dual effects in central nervous system tumors, particularly glioma. On one hand, dysregulated apoptotic pathways in gliomas can contribute to the anti-apoptotic properties of tumor cells, thereby facilitating tumor growth and resistance to treatment (8, 9). Over-expression of anti-apoptotic proteins, such as members of the Bcl-2 family, by glioma cells potentially inhibits the activation of RCD (10, 11). Moreover, the abnormal regulation of RCD pathways can make glioma cells resistant to conventional therapies such as RT and chemotherapy (12). However, in certain scenarios, RCD can have an inhibitory impact on glioma. Studies have indicated that by enhancing RCD in glioma cells, tumor volume can be reduced and treatment efficacy can be improved (13, 14).

Despite the progress made in the realm of RCD, the journey toward its clinical application remains challenging. To date, therapeutic strategies harnessing apoptosis have only yielded a limited number of approved pharmacological agents for human disease management. No agents that intentionally inhibit apoptosis have been established for clinical use. An example of an approved therapy is Venetoclax, a BCL2 inhibitor which has demonstrated efficacy as a monotherapy or in combination regimens for treating chronic lymphocytic leukemia (CLL), small lymphocytic lymphoma (SLL), and acute myeloid leukemia (AML) (15–17). Furthermore, the caspase inhibitor Emricasan garnered accelerated attention from the U.S. FDA in 2016 for its focus on non-alcoholic steatohepatitis (NASH); nevertheless, the clinical outcomes have exhibited variability, underscoring the unpredictable nature of current interventions (18, 19). Looking ahead, a comprehensive investigation into the molecular intricacies of cell death, particularly within the context of the interconnected networks among various RCD pathways, is expected to stimulate innovative breakthroughs. It is through such a thorough understanding of the interplay between these pathways that novel therapeutic strategies can be developed, paving the way for their translation from laboratory research to practical application in medical treatment. The emphasis on these mechanistic insights holds great promise for unveiling new opportunities for clinical intervention and represents a significant advancement in harnessing RCD for disease management.

**Figure 1:**
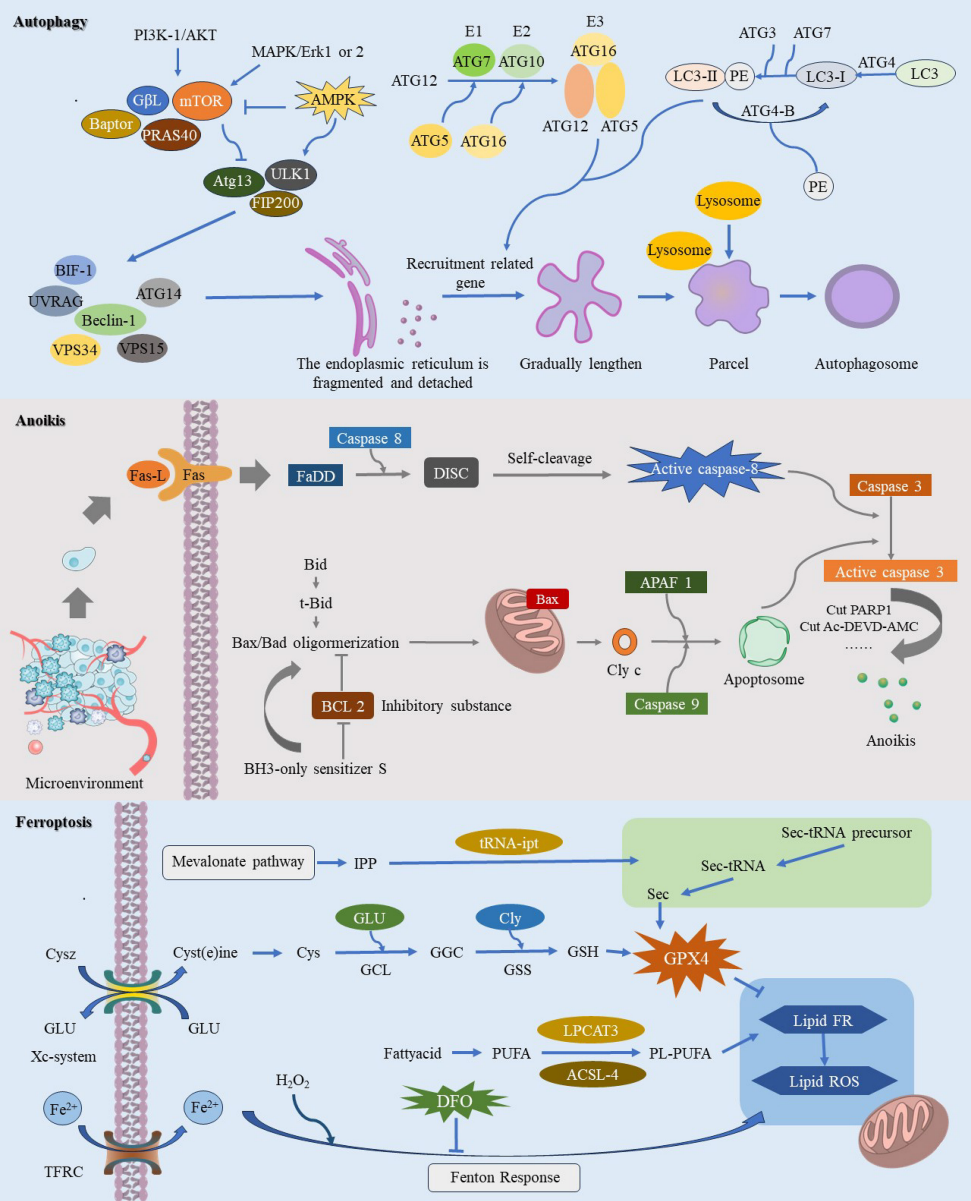
This image provides a comprehensive illustration of the molecular mechanisms underlying three pathways of regulated cell death (RCD): autophagy-dependent cell death, anoikis, and ferroptosis. Autophagy-dependent cell death is characterized by the crucial role of autophagy in degrading essential cellular components, ultimately leading to cell demise. During autophagy-dependent cell death, continuous autophagic activity undermines cellular structure and function, resulting in eventual cell death. Anoikis is primarily initiated when cells dissociate from the extracellular matrix, causing them to lose vital survival signals. This detachment disrupts integrin and other adhesion molecule signaling pathways, leading to the downregulation of survival pathways such as PI3K/Akt and Ras/MAPK, while upregulating pro-apoptotic factors like Bim and Bid. The resulting alterations in mitochondrial membrane potential facilitate the insertion of pro-apoptotic proteins Bax and Bak into the mitochondrial membrane, which then releases cytochrome c and activates downstream apoptotic caspases, ultimately driving cell apoptosis. The process of ferroptosis is initiated by elevated intracellular iron levels, which amplify the Fenton reaction and enhance ROS production. This process triggers lipid peroxidation of polyunsaturated fatty acids in the cell membrane, leading to oxidative stress and compromising membrane integrity, ultimately resulting in cell death.

**Figure 2:**
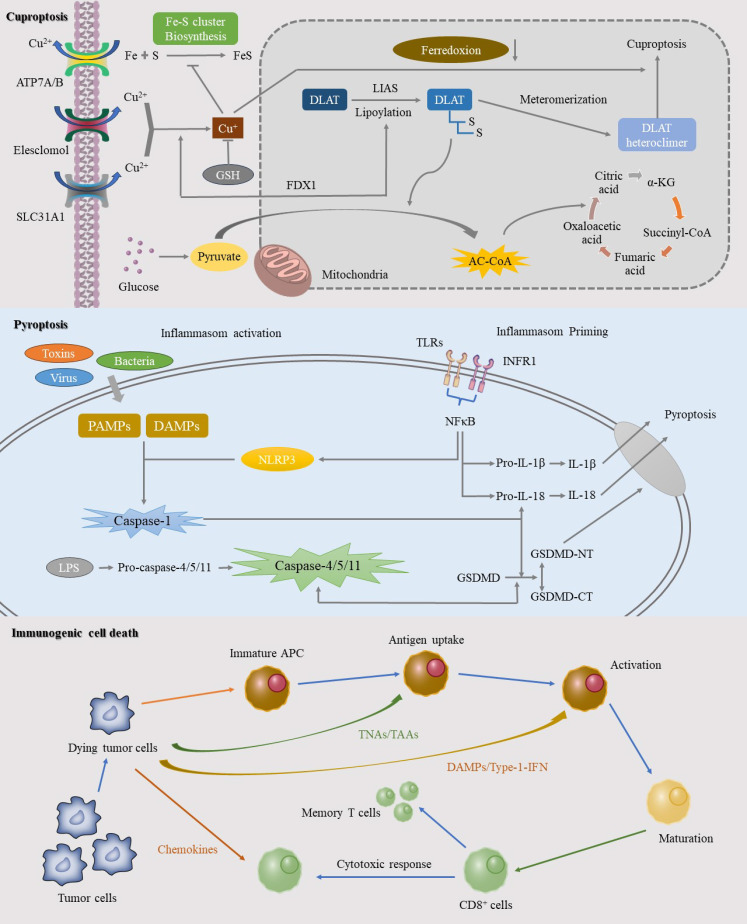
This image elucidates the specific mechanisms of three significant RCD pathways: cuproptosis, pyroptosis, and ICD. Cuproptosis, a copper-dependent form of cell death, is initiated by the intracellular accumulation of copper ions. The mechanism involves the binding of copper ions to thioredoxin cluster proteins, leading to protein aggregation and mitochondrial dysfunction. Pyroptosis, another RCD process, is mediated by inflammasome activation in response to pathogens or intracellular danger signals, resulting in a robust inflammatory response. ICD represents an RCD pathway that triggers a potent anti-tumor immune response. This process involves the release of specific death signals and DAMPs that activate the immune system.

Overall, the regulation of RCD in glioma involves intricate signaling pathways and regulatory factors. Its influence on glioma depends on the complex interplay of multiple factors, including tumor cell characteristics, the tumor microenvironment, and treatment modalities (20, 21). Thus, this review provides a concise overview of the role of RCD in the onset, treatment, and prognosis of glioma. It focuses on several significant apoptotic pathways including autophagy-dependent cell death (22, 23), anoikis (24), ferroptosis (25, 26), cuproptosis (27), pyroptosis and immunogenic cell death (**Figures 1**, **2**; **Table 1**). We propose strategies to optimize and coordinate traditional or novel approaches for glioma treatment based on these pathways. This comprehensive understanding of RCD in glioma will not only enhance readers’ comprehension of its underlying mechanisms but also serve as a theoretical foundation for developing more effective treatment strategies.

**Table 1:** Comparison of distinct aspects of RCD pathways.

RCD pathway	Morphological characteristics		Occurrence mechanism	Common inspection methods and indicators	Major associated disease
Autophagy-dependent cell death (22, 23, 28)	Cytomembrane	The autophagosome is formed, which wraps around and encloses the organelles to be degraded.	The autophagy pathway primarily occurs in two ways (1): non-selective autophagy, involving the extensive degradation of encapsulated organelles and intracellular proteins; and (2) selective autophagy, targeting the degradation of specific entities such as diseased cells or viruses.	The localization of the transcription factor TFEB, determination of ATG expression (such as Atg5, Atg7, and Atg8/LC3), and observation of autophagosomes (including their membrane structure and proteins) are necessary.	Tumors, neurodegenerative diseases, cardiovascular diseases, etc.
	Cytoplasm	The number of autophagosomes gradually increased, and the fusion of lysosomes led to the formation of autolysosomes.			
	Nucleus	No special change.			
	Organelle	The shape and number of mitochondria are significantly altered, and the number of autophagosomes increases, while the structure of the endoplasmic reticulum also appears to be expanded or fragmented.			
Anoikis (24, 29)	Cytomembrane	The cytomembrane displays folds and bulbous expansions, leading to the formation of flower-like ring protrusions.	The loss of cell-matrix force or the weakening of cell-cell adhesion leads to the activation of signaling proteins and apoptosis pathways.	Apoptosis-related markers, such as Annexin V, and nuclear and chromosomal histochemical staining were detected.	It is closely related to the metastasis of tumor
	Cytoplasm	The organelles gradually compact, resulting in cell shrinkage and clumping.			
	Nucleus	Chromatin aggregation, nuclear membrane rupture, and nucleus fragmentation occur.			
	Organelle	The cytoskeleton is reorganized, mitochondrial function is impaired, and ultimately, the system collapses completely.			
Ferroptosis (25, 26)	Cytomembrane	Lipid per, leading to the rupture of cell membranes.	The main cause is the excessive presence of free iron ions in the cell, which leads to oxidative stress and increased lipid peroxidation of the cell membrane. This triggers the signaling pathway for cell death.	The degree of lipid peroxidation, the content of endogenous iron ions, and the expression levels of related genes and proteins were assessed.	It is mainly associated with neurodegenerative diseases and tumors.
	Cytoplasm	Mineralized clusters appear.			
	Nucleus	No special change.			
	Organelle	Mitochondrial structural changes, increased oxidative stress, and endoplasmic reticulum stress.			
Cuproptosis (27)	Cytomembrane	Multiple villous bulges are formed on the cytomembrane	Intracellular overload of copper ions leads to oxidative stress, disrupts the intracellular REDOX balance, and ultimately results in cell death.	The concentration of copper ions, oxidative stress and cell death were measured.	Neurodegenerative diseases and metabolic related diseases.
	Cytoplasm	The organelles become smaller and tightly packed.			
	Nucleus	Nuclear morphology is irregular and nuclear fragments appear.			
	Organelle	Mitochondrial structural changes, increased oxidative stress, and endoplasmic reticulum stress.			
Pyroptosis (30)	Cytomembrane	The cell membrane forms numerous holes, leading to an ion imbalance that causes the cell to swell and dissolve, ultimately leading to the rupture of the cell membrane.	The main way to activate Caspase-1 or Caspase-4/5/11 by inflammasome complexes (such as NLRP3, AIM2), these Caspases then cleave Gasdermin D (GSDMD), causing its N-terminal fragment to insert into the cell membrane and form pores, leading to cell membrane rupture and release of cellular contents, including pro-inflammatory cytokines such as IL-1β and IL-18, thereby triggering a severe inflammatory response and cell death.	Lactate dehydrogenase (LDH) release assay, enzyme-linked immunosorbent assay (ELISA) etc.	Inflammatory diseases, cardiovascular diseases, and tumors.
	Cytoplasm	The cells rapidly swell, and cellular contents leak out, releasing inflammatory factors such as IL-1β and IL-18 into the cytoplasm.			
	Nucleus	The nuclear DNA fragments, but not in a structured way like apoptosis; chromatin is partially condensed.			
	Organelle	Mitochondrial depolarization, loss of integrity of lysosomal membrane.			
ICD (31)	Cytomembrane	The surface exposes high mobility group box protein B1 (HMGB1), calreticulin (CRT), and heat shock proteins (HSPs).	This mainly involves the translocation and release of specific signaling molecules, such as endoplasmic reticulum stress-induced calreticulin (CRT) on the surface and high-mobility group box 1 (HMGB1) released extracellularly, as well as ATP released, which are recognized and taken up by dendritic cells and other antigen-presenting cells.	Flow cytometry, immunofluorescence staining, ATP release detection, etc.	Tumors, autoimmune diseases, and infections.
	Cytoplasm	Releases multiple immune stimulating substances.			
	Nucleus	DNA breaks and chromatin condensation resemble apoptosis, but also involve the release of immune-related molecules.			
	Organelle	The mitochondrial membrane potential is lost, and there is a significant endoplasmic reticulum stress response.			

The divergence in the mechanisms underlying different RCD pathways can be attributed to the regulation of both internal and external signals. These signals encompass DNA damage, perturbation of cell cycle control, cytokine signaling, nutrient deprivation, as well as extracellular matrix cues. Upon sensing these signals, specific pathways are triggered, ultimately leading to cell death. In this table, we compare various RCD pathways in terms of cell morphological characteristics, pathogenesis, commonly employed examination methods and indicators, as well as key associated diseases. By doing so, we aim to provide a comprehensive summary of the commonalities and distinctions among different pathways, which could facilitate future practical applications.

## 2 Glioma and glioma immunotherapy

Gliomas represent 40-60% of primary central nervous system tumors in adults and are the most prevalent primary intracranial tumors in this population. According to the World Health Organization (WHO), gliomas are classified into four grades: grades 1 and 2 are low-grade, whereas grades 3 and 4 are high-grade (32). Despite advancements in glioma treatment over the past decades, challenges persist due to their unique location and the characteristics of “cold” tumors, which contribute to high recurrence rates, poor prognoses, and short median survival times (33).

The immune system fulfills three critical roles: immune surveillance, immune defense, and immune homeostasis, all of which are integral to tumor initiation, progression, metastasis, and response to treatment. Immune checkpoint inhibitors (ICIs) have revolutionized cancer therapy in recent years. However, gliomas, characterized as “cold” tumors, often exhibit poor or non-responsive behavior to ICIs, limiting the success of immunotherapy. Enhancing the immune microenvironment to convert “cold” tumors into “hot” ones may provide a new avenue for improving treatment responses (34, 35).

Bevacizumab, an FDA-approved monoclonal antibody targeting vascular endothelial growth factor (VEGF), inhibits tumor vascularization rather than directly targeting T cells (36). PD-1 inhibitors such as Pembrolizumab and Nivolumab have shown promise in clinical trials. PD-1, a receptor on T cells, interacts with PD-L1 to suppress T cell activity, aiding tumor immune evasion. Pembrolizumab disrupts this interaction, reactivating T cells to recognize and attack tumor cells (37, 38). Similarly, Nivolumab restores T cell functions by blocking the PD-1 receptor. Although a phase III clinical trial comparing Nivolumab plus RT to TMZ plus RT in newly diagnosed patients with non-methylated MGMT promoter gliomas did not achieve the primary endpoint of extending overall survival, it marked a significant step towards using Nivolumab with RT in glioma treatment, without identifying major safety concerns (39). Other immunotherapy strategies are also being explored. Oncolytic viruses (OVs) selectively replicate within and induce apoptosis in cancer cells while sparing normal tissues. A multicenter study using the oncolytic virus DNX-2401 in combination with an anti-PD-1 monoclonal antibody demonstrated safety and survival benefits (40). Dendritic cell (DC) vaccines like DCVax-L hold promise in clinical trials; these vaccines involve extracting and loading dendritic cells with tumor antigens *in vitro* before re-injecting them into the patient to stimulate an immune response (41).

The Nobel Prize in Medicine was awarded to James Allison and Tasuku Honjo six years ago for their groundbreaking work in immunotherapy (42). Current standard treatment for glioblastoma includes surgical resection combined with TMZ chemotherapy but remains insufficient. Recent studies indicate that RCD can play a synergistic role in anti-tumor immunotherapy, including for ICI-resistant tumors. Existing research highlights significant distinctions in RCD processes between low-grade and high-grade gliomas. In low-grade gliomas, apoptosis levels are relatively low, and the role of autophagy remains controversial, though these tumors generally exhibit better cellular survival activity. By contrast, high-grade gliomas demonstrate a higher resistance to apoptosis and possess a more intricate autophagy function, often accompanied by more extensive necrotic areas. These malignant tumors feature markedly different gene expression profiles, which in turn lead to varied treatment responses. Low-grade gliomas tend to be more responsive to radiation and chemotherapy, while high-grade gliomas exhibit a stronger resistance to these treatments (43). As immunotherapy advances, it becomes crucial to investigate the different pathways that either inhibit or activate various RCD processes, as well as their synergistic effects. Such exploration could pave the way for more effective targeted treatments for gliomas.

## 2 Regulated cell death and glioma

### 2.1 Autophagy-dependent cell death

Coined by Christian de Duve and colleagues in 1963, the term “autophagy” describes the process by which cellular components, whether originating from within or outside the cell, are transported to lysosomes for degradation (44). Since the 1990s, research on autophagy has been greatly expanded due to its use as a model organism in yeast studies. Initial findings showed an accumulation of autophagosomes and lysosomes in dying cells, leading to the naming of “autophagic cell death” (45, 46). Autophagy plays multifaceted roles throughout disease progression, particularly in tumor. In the early stages of oncogenesis, autophagy acts as a guardian of genomic integrity and internal stability by regulating quality control and responding to oxidative stress. This helps prevent tissue damage, inflammation, and ultimately inhibits tumorigenesis and metastasis. However, during advanced stages of tumor, autophagy can serve as a source of nutrients that facilitate neoplastic growth, enhance immune evasion, and promote tumor advancement (47). Historically, reliance on morphological delineation has been the cornerstone of autophagy research, resulting in a gap in establishing causal relationships between autophagy and cell death. The present consensus among scientists categorizes autophagy’s involvement in cell death into three primary types: autophagy-related, autophagy-dependent, and autophagy-mediated cell death (48). Autophagy-dependent cell death is considered as a regulated form of cell death that relies on the autophagic apparatus and generally adheres to the following criteria (1): elevated autophagy flux occurring simultaneously with cell death (2), reversibility of cell death when autophagy is inhibited through pharmacological or genetic means (3), dependence on at least two components of the autophagic process, and (4) absence of concurrent alternative forms of cell death (6).

The notion of autophagy-dependent ferroptosis emerged amidst deepening investigations into ferroptosis, a form of cell death described in 2012 as iron-dependent and distinct from apoptosis, autophagy, and necrosis (49). However, the specificity of this classification has come under scrutiny. Because Minghui Gao et al. found that under the action of ferroptosis activator, autophagosomes gradually accumulated, and cells died because of some components of the autophagy mechanism, they named this death mode as autophagy dependent ferroptosis (50–52). This reevaluation highlights the dynamic and interconnected nature of autophagic processes and cell fate, keeping autophagy at the forefront of contemporary biomedical research.

As far as the current research on glioma is concerned, most of the research still stays on the acceleration or delay of the autophagy process. Celastrol, a triterpenoid compound derived from Tripterygium wilfordii, a traditional Chinese medicine, demonstrates potential anti-glioma effects. It activates the ROS/JNK signaling pathway while inhibiting the Akt/mTOR pathway, leading to G2/M phase arrest and triggering autophagy (53). Thiolidazine enhances P62-mediated autophagy by upregulating AMPK activity and the Wnt/β-catenin signaling pathway, thereby inhibiting glioma cell proliferation and migration (54).

In this case, there are still a few studies to explore the relationship between autophagy dependent cell death and the occurrence and development of glioma. Investigator Svenja Zielke established a rigorous criterion for autophagy-dependent cell death and carefully identified three effective agents - loperamide, pimozide, and STF-62247 - from a wide range of over 70 compounds that induce autophagy. These agents were observed to promote LC3B lipidation and puncta formation, which are hallmarks of autophagic activity. This promotes autophagic flux through interactions with essential autophagy proteins ATG5 and ATG3. Ultrastructural analysis revealed an abundance of autophagosomes and autolysosomes in cells treated with loperamide and pimozide. Additionally, these compounds induced a noticeable dephosphorylation trend in complex 1 of the mammalian targets of rapamycin (mTOR), indicating activation of autophagy. Consequently, these findings suggest that loperamide, pimozide, and STF-62247 could potentially trigger autophagy-dependent cell death in glioma cells, providing a novel paradigm for therapeutic intervention in glioblastomas (55). Separately, amentoflavone (AF), a polyphenolic compound endemic to the Selaginella species, exhibits a broad spectrum of biological activities including anti-inflammatory, anti-neoplastic, radioprotective, antioxidative, and neuroprotective properties. Experimental observations have documented conspicuous depletion in intracellular glutathione levels and mitochondrial membrane potential following AF exposure, concomitant with a surge in cellular iron concentrations, malondialdehyde, and lipid peroxidation. Subsequent inquiry has substantiated the capability of AF to inhibit neoplastic proliferation by instigating autophagy-dependent ferroptosis *in vivo* potentially attributable to the modulation of the AMP-activated protein kinase (AMPK)/mTOR signaling cascade (56). These insights could delineate unrecognized molecular conduits for curtailing tumor growth and reinforce the therapeutic potential of autophagy-centric interventions in tumor management.

### 2.2 Anoikis

When a cell loses its normal connection to the stroma, it senses signaling abnormalities that trigger anoikis, leading to disruption of intracellular signaling and cell death. Ultimately, this results in the effective removal of displaced cells (29). Because of this characteristic, this RCD pathway has been named amnesiotic apoptosis. NCCD defines lost-nest apoptosis as a specific variant of intrinsic apoptosis induced by integrin-dependent anchoring loss (6).

In the case of tumors, the absence of anoikis can promote tumor cell survival and proliferation, thereby facilitating tumor development and drug resistance (57–59).

With advancements in bioinformatics, several researchers have developed predictive models based on genes associated with anoikis. Zhongzheng Sun et al. utilized nine genes related to anoikis to predict the survival rate and prognosis of glioma patients. They constructed a risk score and employed this model to forecast patient outcomes. Based on these findings, the possibility of exploring immunotherapy for glioma using genes and mechanisms correlated with anoikis was investigated (60). Dongdong Zhang analyzed the expression and survival of genes related to five types of anoikis, including ETV4, HMOX1, MYC, NFE2L2, and UBE2C. Additionally, a clinical prediction model was constructed (61). This work laid the foundation for subsequent progress in basic experimental research. The lncRNA ANRIL exhibited a positive correlation with glioma grade in glioma tissues regarding the induction of anoikis, and it was found to indirectly induce anoikis by inhibiting the anti-apoptosis gene Bcl-2 (62). In terms of the tumor microenvironment, MNX1 was found to be ectopically expressed in glioma cells and associated with glioma grade. This substance was observed to contribute to cell adhesion and potentially enhance the ability of glioma cells to evade anoikis by regulating the expression of its downstream molecule TrkB (63).

### 2.3 Ferroptosis

Ferroptosis is a recently discovered novel form of RCD that is dependent on reactive oxygen species (ROS). The process of cell death is typically accompanied by significant accumulation of iron and lipid peroxidation (64). With the deepening of the understanding of ferroptosis, many studies believe that ferroptosis should be attributed to autophagy dependent ferroptosis, but due to the uniqueness of its cause and process, it is still discussed as a separate concept here.

Ferroptosis is intricately linked to various diseases and pathological processes, encompassing neurological conditions (e.g., Parkinson’s disease, Alzheimer’s disease) (65, 66), cardiovascular diseases (67), and liver diseases (68). The orchestrated process of ferroptosis in gliomas is primarily regulated by four pathways, including metabolism, the GPX4 pathway, the FSP1 pathway, and lipid metabolism (69). In the process of iron metabolism, cells absorb ferric ions (Fe3+) via the transferrin receptor TfR1, and subsequently reduce them to ferrous ions (Fe2+). The GPX4 pathway represents the classical pathway of ferroptosis. At the heart of this pathway is the lipid repair enzyme glutathione peroxidase 4 (GPX4), which plays a crucial role in inhibiting intracellular lipid peroxidation by degrading small molecule peroxides and some lipid peroxides using GSH as a substrate (70).

Current studies have provided evidence that various factors and pathways can either induce or inhibit ferroptosis in glioma. Strychnine, a weakly basic indole alkaloid derived from strychnine seeds, has demonstrated potent antitumor activity against multiple tumor types, including glioma (71). Recent investigations have revealed its ability to induce ferroptosis in glioma cells (72). TRIM7, which utilizes a K4-linked chain, directly binds to and ubiquitinates nuclear receptor coactivator 4 (NCOA4), thereby reducing NCOA4-mediated ferritin phagocytosis and subsequent ferroptosis in human glioblastoma cells (73). Ferroptosis exhibits both beneficial and adverse effects in the development and progression of glioma. Some studies have suggested that increased ferroptosis may compromise the efficacy of PD-L1 immunotherapy, while inhibiting ferroptosis remodels the immunosuppressive microenvironment and enhances the effectiveness of immunotherapy (74). Conversely, plumbagin has exhibited tumor growth inhibition by targeting the NQO1/GPX4 pathway-mediated ferroptosis, suggesting therapeutic implications for inducing ferroptosis (75). In summary, ferroptosis exhibits dual effects on glioma, influencing the efficacy of immunotherapy and potential therapeutic strategies. However, a comprehensive understanding of ferroptosis in gliomas remains elusive, necessitating further research to identify key molecular targets and mechanisms.

### 2.4 Cuproptosis

Similar to ferroptosis, cuproptosis is a copper-dependent and unique cell death (76). A recent study suggests that cuproptosis is an independent form of RCD and is highly associated with mitochondrial respiration and the lipoic acid (LA) pathway (77), and plays a key role in tumor cell proliferation, metastasis, and drug resistance.

Studies on cuproptosis in glioma have mainly relied on bioinformatics and gene sequencing techniques for which there are limited experimental validations available. Due to the restricted predictive value of individual biomarkers, researchers have incorporated multiple biomarkers into models to predict the behavior and progression of glioma accurately. For example, Bo Chen et al., identified a signaling pathway along with 13 ligand-receptor pairs such as ICAM8, ITGAX, ITGB1 ANXA2-FRR1 etc., constructing an activity score based on genes associated with cuproptosis while ensuring its stability through machine learning technology (78). Similarly, Lin Wang et al. identified 10 lncRNAs associated with cuproptosis that target 7 resistance genes (FDX1, LIAS, LIPT1, DLD, DLAT, PDHA1, and PDHB) and 3 sensitization genes (MTF1, GLS, and CDKN2A). They developed a prognostic risk model based on these cuproptosis-related lncRNAs which accurately predicts the tumor microenvironment status (79). Notably, FDX1 has been found to be highly expressed in gliomas and is primarily involved in lipid acylation of tumor proteins and cuproptosis, which significantly affects the prognosis of low-grade gliomas (80). Methylation of FDX1 is believed to promote malignant behavior in glioma. Functional experiments further revealed that the target gene C-MYC enhances the proliferation and invasion of glioma cells through YTHDF1 and FDX1 methylation, possibly due to aberrant copper ion behavior in mitochondria (81). Although existing studies are insufficient to establish the clinical value of cuproptosis-related mechanisms in glioma development and progression, multiple potential factors indicate that cuproptosis, like other RCD modalities, warrants further investigation and can profoundly impact glioma research.

### 2.5 Pyroptosis

Pyroptosis, a newly identified form of RCD, plays a pivotal role in the body’s defense against pathogens. This mechanism is distinguished by specific morphological changes, including cell swelling, vesicle formation, membrane perforation, and eventual cell lysis. Unlike ferroptosis and autophagy, cell pyroptosis is mediated by proteins of the gasdermin (GSDM) family, which cause progressive cell swelling until membrane rupture. This process is tightly linked to inflammatory cascades and immune responses and can be categorized into classical and non-classical types based on the activation of caspase-1 (82).

To date, most investigations into pyroptosis in glioma have been conducted at the bioinformatics level, with limited experimental and clinical validation. Guilong Tanzhu and colleagues developed a prognostic model leveraging lncRNAs associated with pyroptosis to predict glioma patient outcomes. They further analyzed molecular alterations and immune infiltration across different risk groups, constructing a lncRNA-miRNA-mRNA regulatory network grounded in prognostic gene risk scores (83). Hanzhang Liu et al. identified nine key differential genes by examining 523 low-grade glioma (LGG) and 1,152 normal tissue samples. Their findings indicate that these genes are instrumental in LGG progression and tumor immunity, potentially benefiting prognosis and immunotherapy (84). Caspase-6 (CASP6), a critical protein in the pyroptosis pathway, presents fluctuating relevance in glioma prognosis. Kai Guo et al. conducted an extensive analysis of four bioinformatics data routes, extracting pyroptosis-related differentially expressed genes (PRDEGs) from 81 data sets within the GeneCards database. Their research revealed CASP6 overexpression in glioma, primarily implicating it in immune responses and antigen processing. CASP6 expression was inversely correlated with overall survival and disease progression in glioma patients (85).

In summary, bioinformatics analyses suggest a significant link between pyroptosis and glioma pathogenesis. Future studies encompassing basic experimental validation and clinical research are crucial to elucidate the precise mechanisms and impacts of pyroptosis in glioma.

### 2.6 Immunogenic cell death

Prior to the identification of ICD, apoptosis was typically regarded as a non-immunogenic process. The recent definitions by the Committee on Cell Death Nomenclature (NCCA) describe ICD as a regulated form of cell death that can activate host immune responses within an adaptive immune context. Unlike other forms of cell death, ICD is triggered by a narrow range of stimuli, including chemotherapy agents, viral infections, and various physical or chemical factors. These stimuli induce a cascade of damage-associated molecular patterns (DAMPs), subsequently eliciting an immune response (6).

Curcumin, a bioactive phenolic compound, exhibits antioxidant, anti-inflammatory, and antibacterial properties, and has been documented to modulate several cell death pathways. Research conducted by Zenghe Xiu et al. demonstrated that curcumin can potentiate ionizing radiation-induced death of glioma cells via activation of the endoplasmic reticulum (ER) stress PERK-eIF2α and IRE1α-XBP1 signaling pathways—a finding corroborated in murine models (86). Carbonic anhydrase IX (CAIX) is a tumor-associated cell surface glycoprotein that moderates tumor cell survival by influencing pH homeostasis. S4, a highly selective CAIX inhibitor, has shown substantial efficacy in breast and colorectal cancer models. Investigations by Jing Cui et al. revealed that S4 markedly diminishes glioma cell viability and induces both apoptosis and autophagy. Further analysis indicated that S4 promotes calreticulin exposure and the release of HMGB1 and HSP70/90, thereby activating the PERK-eIF2α and IRE1α-XBP1 pathways and facilitating DAMP release linked to ICD in glioma cells through the ER stress pathway (87). Recent studies assessed the potential of TNFAIP2 knockout in enhancing the therapeutic outcomes of PD-1 inhibitors. Both *in vitro* and *in vivo* experiments demonstrated that TNFAIP2 knockout increases surface expression of calreticulin (CALR), heat shock protein 70 (HSP70), and heat shock protein 90 (HSP90) in glioblastoma (GBM) cell lines, thereby inducing ICD. Consequently, TNFAIP2 knockout during PD-1 therapy could significantly bolster survival rates in glioma patients (88).

While molecular mechanisms underlying ICD have been extensively studied, the clinical implications of ICD in disease classification, treatment, and prognosis remain underexplored. Bioinformatics offers a robust platform to address these challenges. Jiayang Cai et al. identified 34 ICD-related genes, ultimately focusing on 12 key genes including IL17RA, IL1R1, EIF2AK3, CD4, PRF1, CXCR3, CD8A, BAX, PDIA3, CASP8, MYD88, and CASP1. They established associations with non-codeletion of 1p19q, higher WHO grades, wild-type IDH, and immunosuppressive tumor microenvironments (89). Additionally, Sun et al. analyzed 1,896 glioma samples across five databases, devising a risk score model based on 14 ICD-associated genes that independently predicts survival and response to immunotherapy in glioma patients (90).

### 2.7 Crosstalk between multiple RCDs

Different modes of RCD, including apoptosis and necroptosis, have been extensively studied and elucidated. Apoptosis, characterized by its orderly and systematic nature, is vital for maintaining tissue homeostasis and eliminating damaged cells, thereby influencing numerous diseases and pathophysiological states. Under physiological conditions, apoptosis ensures the timely demise of cells through the meticulous regulation of both intrinsic and extrinsic signaling pathways (91). Glioma cells frequently demonstrate overexpression of anti-apoptotic proteins (e.g., Bcl-2, Bcl-xL) and deactivation of pro-apoptotic proteins (e.g., Bax, Caspase family). This apoptosis evasion mechanism not only enhances the survivability of tumor cells but also increases their resistance to conventional chemoradiotherapy and chemotherapy (92). Hence, restoring the normal apoptotic pathway is a pivotal research direction in anti-glioma therapies, with the reactivation of apoptosis presenting a promising strategy for effective tumor cell eradication. Necroptosis, a form of RCD that intersects characteristics of both apoptosis and necrosis, is primarily regulated by the receptor-interacting serine/threonine-protein kinases 1 and 3 (RIPK1 and RIPK3). In contrast to apoptosis, necroptosis resembles necrosis through the rupture of the cell membrane and the release of pro-inflammatory factors (93). Glioma cells often manipulate the necroptosis pathway to avert cell death, including the inhibition of RIPK1/RIPK3 activity and the prevention of downstream mixed lineage kinase domain-like protein (MLKL) translocation (94). The induction of necroptosis via exogenous signals such as tumor necrosis factor (TNF) or chemotherapeutic agents can lead to irreversible cell death in glioma cells, coupled with a potent inflammatory response that augments the immune system’s ability to target tumor cells. However, the excessive activation of necroptosis can also precipitate unnecessary tissue damage and inflammation, underscoring the necessity of precise regulation and the establishment of an optimal therapeutic window. Overall, a deeper understanding of necroptosis in glioma could pave the way for the development of innovative targeted therapies, thereby enhancing patient outcomes.

Tumors exhibit intricate crosstalk among various RCD pathways. Despite mechanistic and regulatory differences, these pathways can interact and influence one another under certain conditions, highlighting the importance of intercellular communication, especially as tumor cells develop mechanisms to evade drug-induced apoptosis in the context of targeted therapies.

Pyroptosis and apoptosis are primarily interconnected through the caspase family. The caspase-3/GSDME axis acts as a switch between these two forms of cell death, significantly impacting conditions like lung cancer and melanoma (95, 96). In infectious disease contexts, ZBP1 functions as a sensor for NLRP3 inflammasome activation triggered by influenza virus, paralleling the role of caspase-11 or caspase-4/5 in the non-classical LPS-induced pathway, intertwining with both pyroptosis and apoptosis. Additionally, ZBP1 is crucial in initiating PANoptosis in response to IAV (97).

The tumor suppressor gene TP53 mediates apoptosis and ferroptosis. TP53 upregulates numerous pro-apoptotic genes including BAX, PUMA, and NOXA, promoting mitochondrial-mediated apoptosis by inducing mitochondrial outer membrane permeabilization, releasing cytochrome c, and activating downstream caspases. TP53 also suppresses the SLC7A11 gene, which encodes a component of the system Xc^- transmembrane amino acid transporter responsible for importing glutathione precursor cysteine. Consequently, inhibition of SLC7A11 by TP53 reduces glutathione synthesis, leading to lipid peroxide accumulation and inducing ferroptosis (98). Ferroptosis is characterized by ROS generation and lipid peroxidation, processes that intersect with endoplasmic reticulum (ER) stress. Under ER stress, transcription factors such as IRE1, PERK, and ATF6 are activated, regulating apoptosis-related proteins like Bcl-2 and cytochrome c, thereby influencing the apoptosis pathway. Thus, modulating ferroptosis can also impact apoptosis (99).

Autophagy, particularly lipid autophagy and Beclin-1-mediated xCT degradation, can precipitate ferroptosis. Recent research has revealed that autophagy can induce ferroptosis through the regulation of intracellular ROS, LIP, and lipid peroxide levels, defined as autophagy-dependent ferroptosis (52). Studies by Eunhee Park et al. demonstrated that autophagy could be induced by ROS generated by erastin, an inducer of ferroptosis, with ferritin degradation and increased transferrin receptor 1 (TfR1) expression leading to ferroptosis. NRF2 plays a pivotal role in both ferroptosis and necroptosis; it can activate NLRP3 and AIM to induce necroptosis and exhibits specific functions in ferroptosis. Contrarily, NRF2 can also inhibit NLRP3 activation by reducing ROS levels, thereby blocking necroptosis (100, 101).

ICD is distinctive for its capacity to elicit an immune response. Although distinct from apoptosis, programmed necrosis, autophagy, and ferroptosis, these pathways can transition into ICD under specific conditions. Research on processes such as copper-induced cell death and aneuploid apoptosis in gliomas remains limited, with insufficient evidence to confirm their interaction with other RCD pathways. Collectively, the interrelationships among various RCD pathways are complex and tightly knit, forming an interactive network that regulates cell death and immune responses. Understanding these relationships is paramount for advancing treatments for tumors, autoimmune diseases, and infectious diseases.

## 3 The promise of RCD in targeted glioma therapy

### 3.1 Potential application of autophagy-dependent cell death in glioma targeted therapy

Autophagy-dependent cell death is a type of RCD that relies on autophagy mechanisms or components and plays a significant role in the central nervous system (28). Despite extensive research, there is still no definitive causal link between autophagy and cellular demise. The interplay between autophagy and various forms of cell death makes it challenging to distinguish them based solely on morphological observations throughout the cellular lifecycle. Therefore, here we mainly discuss the therapeutic concepts and methods based on inhibiting or accelerating the autophagy process to hinder tumor development.

The underlying mechanisms of autophagy that contribute to cellular death are notably distinct from those that promote cellular survival. These differences are primarily characterized by variations in the rate of autophagic flux, the duration and intensity of the involved pathways, as well as the nature and turnover rates of the substrates undergoing autophagic recycling and degradation. Such nuances play a crucial role in determining the balance between cell viability and death, thus representing potential focal points for innovative tumor therapies. A wealth of preclinical research has meticulously examined the function of autophagy in tumor treatment, employing two prevalent methodologies. The first method involves triggering autophagy through the administration of broad-spectrum anticancer agents, such as rapamycin, which antagonizes mTORC1 (102). The second strategy involves indirectly activating autophagy by selectively inhibiting specific drug targets, such as the inhibition of ERK in PDAC cell lines (103). Although the precise mechanism underlying modulation of autophagy remains elusive, the current understanding leans towards the notion that upregulation of autophagy confers a protective effect on tumor cells.

With the increasing understanding of autophagy, novel therapeutic agents targeting glioma cell autophagy have entered clinical trials. Chloroquine (CQ) and hydroxychloroquine (HCQ), known for their anti-malarial properties, have shown potential in inhibiting autophagy and enhancing the efficacy of DNA damage therapy. Previous studies investigated combination therapy with hydroxychloroquine for glioma by conducting phase I/II clinical trials to determine its efficacy and maximum tolerated dose (MTD). Despite effectively inhibiting autophagy, the clinical application of hydroxychloroquine is limited due to its toxicity at higher dosages (104). After that, while HCQ has been widely used in the treatment of malaria (105), primary progressive multiple sclerosis (106), IgA nephropathy (107), and even COVID-19 (108), it has rarely been utilized for glioma. However, in 2020, due to the COVID-19 pandemic and the urgent need for new glioma treatments, HCQ and CQ have regained attention, leading to the emergence of corresponding clinical trial studies. In a recent study by Inge Compter et al., the investigators examined the safety, pharmacokinetics, and MTD of a combination therapy consisting of chloroquine, RT, and daily TMZ administration in newly diagnosed glioblastoma patients. This novel therapeutic approach holds promise for the treatment of gliomas (109).

Furthermore, due to the gradual advancement of autophagy research and the rapid progress in bioinformatics in recent years, numerous studies have progressed to the stage of clinical trials. Mutations in the epidermal growth factor (EGF) have been implicated in gliomas’ resistance to conventional chemotherapy (110). Chloroquine, a potent autophagy inhibitor, impairs this survival mechanism by blocking the lysosomal degradation pathways activated by mutated epidermal growth factor receptor (EGFRvIII). This recently registered clinical study marks a new frontier in efforts to overcome therapeutic resistance within gliomas. A pioneering Phase I/II clinical trial, initiated in 2020, is investigating the combined efficacy of dabrafenib, trametinib, and hydroxychloroquine in the treatment of recurrent gliomas characterized by BRAF mutations. This trial specifically focuses on determining the extent of autophagy inhibition and its correlation with resistance profiles of gliomas, as well as the specific involvement of autophagic processes in the context of BRAF anomalies. Building on preclinical findings, delta-9-tetrahydrocannabinol and cannabidiol (CBD) have demonstrated their potential in tempering glioma proliferation and metastasis. These phytocannabinoids engage type 1 and type 2 cannabinoid receptors, triggering the endoplasmic reticulum stress response and subsequently inducing autophagy (111). Last year witnessed the initiation of a Phase I multicentric clinical trial in Spain dedicated to assessing the therapeutic safety of this novel combined modality. Phase I/II clinical trials are currently underway to investigate sphingoid modulators and the glioma autophagy inducer idroxioleic acid. These studies aim to determine the MTD of these drugs, evaluate their safety and initial efficacy, and shed light on their potential for anti-tumor treatment of glioma (112).

Unfortunately, numerous autophagy-related molecules possess a wide range of vital physiological roles beyond their established functions within the autophagic pathway. Autophagy-related genes (ATGs) exert their influence on various processes, both in healthy and pathological states, that go beyond their canonical duties as autophagy facilitators. For example, the ATG5-ATG12/ATG16L1 protein complex has demonstrated antiviral capabilities mediated by interferon-gamma—actions that differ from its involvement in autophagy (113, 114). Additionally, ATG7 is one of the core proteins of autophagy, but the ATG7 dependent non-autophagy pathway plays a key role in the regulation of neovascularization (115). While the classical autophagy pathway remains an essential aspect of ATG function, the non-autophagic capabilities demonstrated by these molecules cannot be disregarded. The multifaceted nature of autophagy-related molecules often complicates the understanding of their complex physiological interactions, presenting a significant challenge in unraveling their roles in clinical trials, which may contribute to suboptimal outcomes observed. Future research endeavors must adopt a broader perspective that is not confined to the solitary functions attributed to autophagy-related molecules. Enhancing our understanding of their versatile involvement in cell death regulation and the distinct disease-modulating processes mediated through autophagy-dependent death pathways in various neoplastic contexts will be crucial for advancing therapeutics.

### 3.2 Potential application of anoikis in glioma targeted therapy

**Figure 3:**
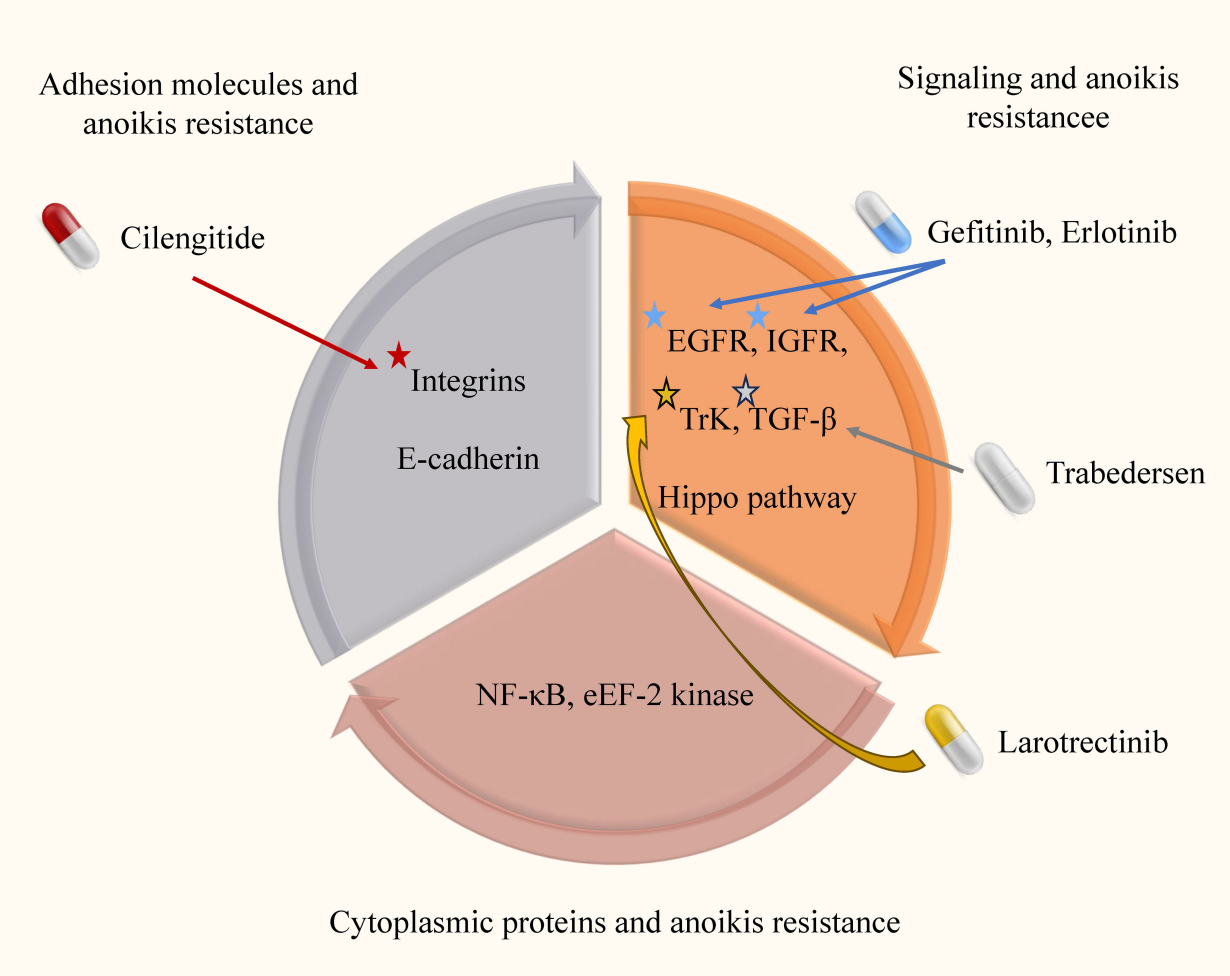
Anoikis resistance mechanism and targeted drugs in glioma. In this image, we elucidate the intricate anoikis-resistance pathways operative in glioma, along with their consequential clinical implications. Anoikis, which is inherently designed to eliminate impaired cells, is crucial for maintaining cellular equilibrium within the microenvironment. However, in gliomas, a disrupted resistance to anoikis emerges, enabling cancerous cells to evade this regulatory process and disrupting the natural course of malignant cell clearance.

Anoikis, a distinct form of RCD triggered by cell detachment, holds significant importance in maintaining cellular homeostasis and microenvironmental stability. Mechanisms that activate resistance to anoikis hinder the induction of apoptosis in cells upon tumor initiation, impeding their ability to undergo cell death when detached. This phenomenon promotes the proliferation and migration of tumor cells. Consequently, inhibiting resistance to anoikis and restoring normal anoikis processes emerge as promising avenues for exploration in tumor research (**Figure 3**).

Despite the inherent radiation resistance of gliomas, RT remains a standard treatment in the context of tumor drug resistance (116). In a study of prostate cancer, Erk and PIK-AKT signaling enabled isolated tumor cells to acquire resistance related to anoikis, leading to drug resistance (117). In glioma, the commonly used alkylating agent TMZ is an essential component of adjuvant chemotherapy. However, recent research has demonstrated that astrocytes resistant to anoikis exhibit robust resistance to TMZ, resulting in minimal reduction in cell viability and unaffected colony formation (118). These findings suggest a potential association between induction of anoikis and drug resistance in glioma.

Considering the potential correlation between anoikis and the development of drug resistance in RT and chemotherapy, targeted therapy against anoikis demonstrates promise as an ideal adjunctive strategy in glioma treatment. Exploring the molecular regulation of anoikis may provide novel insights into prognostic assessment in glioma. For instance, a recent study developed a prognostic model for low-grade glioma (LGG) based on the ANRG and LGG subtypes (119). Another investigation employed differentially expressed anoikis-related genes (ARG) derived from GeneCards to construct a prognostic ARG model for glioma, revealing that patients in the high-risk group exhibit a worse prognosis (61). These findings collectively underscore the clinical significance of anoikis in glioma treatment and prognosis, despite the need for further mechanistic investigations. Continued research in this area will undoubtedly contribute to advancements in glioma therapeutics.

### 3.3 Potential application of ferroptosis in glioma targeted therapy

With the increasing understanding of ferroptosis, researchers have initiated investigations into its potential in tumor therapy. Multiple studies have demonstrated that specific anti-tumor drugs can induce ferroptosis, highlighting its potential in glioma treatment.

Recent findings suggest a close association between ferroptosis and treatment resistance in targeting tumor cells. Viswanathan et al. demonstrated that the lipid peroxidase pathway, mediated by GPX4 which can partially suppress ferroptosis, regulates the development of highly resistant mesenchymal cells (120). Another study revealed that depleting immunosuppressive cells in the tumor microenvironment can reverse drug resistance through ferroptosis (121). These findings underscore the potential of ferroptosis as an effective adjunct therapy against glioma resistance.

Extensive research has been sparked into the potential induction of ferroptosis in tumor treatment, with several clinically approved drugs (e.g., sorafenib and artesunate) demonstrated to effectively induce ferroptosis (122). The integration of nanomaterials with targeted ferroptosis has garnered significant attention in the field of tumor treatment research. Nano-based drug delivery systems offer advantages such as enhanced stability, improved availability, minimal side effects, and robust targeting capabilities. Chen et al. developed trehalose-loaded nanoparticles that combined GSH consumption-induced ferroptosis with trehalose-induced autophagy, demonstrating the potential of nanaterial design in developing ferroptosis-induced nanomedicine (123). While most nanoscale research on ferroptosis has focused on breast and liver cancer, this approach is expected to become a future treatment strategy for glioma. It also demonstrates the importance of interconnection between different RCD pathways.

The increasing recognition of the significance of ferroptosis in the tumor microenvironment and its potential as a prognostic predictor has led to studies investigating its clinical relevance. Zhuo et al. conducted a study examining genetic markers associated with ferroptosis and found that these markers could potentially serve as prognostic indicators for glioma patients (124). Similarly, Wan RJ et al. identified differential expression of ferroptosis-related genes between glioma patients and non-patients, suggesting the possibility of using these genes as a new standard for prognostic evaluation in the future (125). These findings highlight the significance of comprehending the role of ferroptosis in the tumor microenvironment and its potential as a prognostic marker for glioma patients. Further research is necessary to validate and expand upon these findings, ultimately advancing prognostic prediction in glioma.

### 3.4 Potential application of cuproptosis in glioma targeted therapy

Perturbations in serum and tumor tissue copper levels have been observed in patients with malignant tumors such as lung cancer (126) and breast cancer (127), suggesting alterations in copper homeostasis that may contribute to tumor progression, invasiveness, or drug resistance. Although experimental research on cuproptosis is still in its early stages, it is undeniable that cuproptosis holds promising potential as a therapeutic target for tumor treatment.

Pertinent findings from several studies have established a correlation between cuproptosis markers and the prognosis of glioma. In research conducted by Li Wang and colleagues, notably elevated expression levels of cuproptosis-related genes were observed in the high-risk group of glioma patients as compared to the low-risk group. Moreover, the researchers developed a risk model grounded on long non-coding RNA (lncRNA) associated with cuproptosis, which partially reflects its prognostic significance in glioma (79). However, additional experimental validation is necessary to corroborate the prognostic implications of cuproptosis-related genes in this disorder.

In our review of tumors, we have discovered that certain metal ions, namely cadmium (128), chromium (129), and bismuth (130), possess carcinogenic properties to some extent (**Table 2**). Recent research suggests that their mechanism of action may be closely linked to ferroptosis, cuproptosis, and autophagy. Ashish Tyagi et al. conducted mouse experiments and found that chronic exposure to cadmium consistently activates the NOX1 complex, leading to the generation of ROS and endoplasmic reticulum (ER) stress in cells. This activation subsequently results in defective autophagy (131). In a separate study conducted by Caijun Zhao et al., it was discovered that cadmium-induced ER stress mediates the activation of autophagy, which leads to ferroptosis in renal tubular epithelial cells (128). The potential therapeutic application of these metal ions in disease treatment is a topic of discussion, for instance, bismuth has been widely used for the eradication of H. pylori infection (132). It is important to note that the therapeutic and side effects of metal ions are often dose-dependent and influenced by the method of administration. Currently, metal ions have not been utilized in tumor therapy, and further research is necessary to explore their potential in this field.

**Table 2:** Comparative analysis of metal-mediated RCD pathways.

Metal-mediated cell death pathway	occurrence mechanism	Induction pathway	suppressor pathway	clinical application value
Ferroptosis (25)	It mediates cell death by participating in intracellular REDOX reactions and generating ROS.	Iron overload, oxidative stress, and abnormal glutathione metabolism.	Inhibition of iron ionophores and enhancement of antioxidant system.	Could be a potential treatment for tumors.
Cupoptosis (27)	It participates in a series of REDOX reactions within the cell, leading to intracellular oxidative stress that promotes cellular death.	Imbalance and oxidative stress in copper ion tray system.	Not clear.	Could be a potential treatment for tumors.
Cadmium-induced cell death (128)	Cell death is mediated through various mechanisms, such as interfering with the REDOX balance, activating the apoptosis pathway, inducing mitochondrial dysfunction, and causing DNA damage.	Accumulation of cadmium ions, mitochondrial dysfunction.	Reduce the accumulation of cadmium ion and enhance mitochondrial function.	It may influence cell apoptosis, promoting or inhibiting the development of certain tumors.
Chromium-induced cell death (129)	Cell death is mediated by interfering with DNA repair, activating apoptotic pathways, and inducing oxidative stress.	Chromium ion excess, oxidative stress.	Not clear.	Could be a new way to treat tumor.
Bismuth-induced cell death (130)	It mediates cell death by inducing oxidative stress, interfering with mitochondrial function, and triggering apoptosis.	Accumulation of bismuth ions.	Not clear.	Could be a new way to treat tumor.

Metal-induced RCD plays a significant role in cellular demise. In this phenomenon, metal ions exert regulatory control over the apoptotic signaling pathway by interacting with crucial intracellular proteins, ultimately leading to cell death. In the present study, we provide a comprehensive comparison of disparate metal-mediated RCD pathways, focusing on the pathogenesis, induction, and inhibition of these pathways in tumor cells. Furthermore, we discuss the potential clinical applications associated with these metal-induced RCD pathways.

### 3.5 Potential application of pyroptosis in glioma targeted therapy

The relationship between pyroptosis and tumor dynamics, particularly in glioma, is intricate and multifaceted. On one hand, as a significant form of RCD, pyroptosis can effectively inhibit tumor initiation and progression. Conversely, the inflammation and immune responses triggered by pyroptosis release various inflammatory mediators and cytokines, such as interleukin-1β and interleukin-8, which might promote tumor growth, modify the tumor microenvironment, and contribute to drug resistance (133).

Current research endeavors aim to harness pyroptosis to enhance glioma treatment. For instance, Li-Wen Ren utilized weighted gene co-expression network analysis (WGCNA) on data from The Cancer Genome Atlas (TCGA) to identify key genes in glioblastoma (GBM). Using the connectivity map (CMAP) platform, three substances—flubendazole, mebendazole, and fenbendazole—were identified as potential anti-glioma agents. These compounds were shown to induce pyroptosis in GBM cells via the NF-κB/NLRP3/GSDMD pathway and also trigger mitochondria-dependent apoptosis in nude mice models (134). In another study, Wenpeng Zhao and colleagues conducted high-throughput screening of 2,718 compounds from approved drug and clinical compound libraries. They discovered that the second-generation small molecule polyCDK inhibitor AT7519 holds promise as a GBM therapy. AT7519 notably inhibited glioma cell viability and proliferation in a dose-dependent manner and induced pyroptosis through caspase-3-mediated cleavage of gasdermin E (GSDME) (135). Meanwhile, Chinese researchers have explored traditional Chinese medicine formulations, identifying Xihuang Pill as a noteworthy candidate. This traditional preparation promotes the pyroptosis of glioma cells via the POU4F1/STAT3 axis and effectively halts glioma proliferation, although its precise mechanisms and clinical applicability warrant further investigation (136). Despite these promising approaches, translating them into effective clinical treatments faces substantial challenges. The glioma microenvironment is exceptionally complex and exhibits significant heterogeneity in treatment response. Furthermore, researchers must address issues of selectivity, drug delivery, side effects, and resistance associated with pyroptosis-inducing agents.

In summary, while pyroptosis offers potential therapeutic avenues for glioma, extensive research is necessary to overcome the inherent challenges and fully realize its clinical benefits.

### 3.6 Potential application of ICD in glioma targeted therapy

In 1893, American orthopedic surgeon William Coley made a serendipitous discovery that postoperative pyogenic streptococcal infections could induce tumor regression in sarcoma patients, thus marking the dawn of tumor immunotherapy. Over the past decade, ICD has emerged as a crucial link between oncological treatment and the immune system, paving the way for numerous advancements in cancer therapy. Several factors can induce ICD, including chemotherapeutic agents, oncolytic viruses, high hydrostatic pressure, and cytotoxic heat shock (31). ICD inducers can be categorized based on their immune induction mechanisms: Type I inducers primarily comprise genotoxic agents that lead to abnormal protein activation and DNA damage, while Type II inducers are generally more potent, inducing endoplasmic reticulum oxidative stress, perturbing ER homeostasis, raising intracellular Ca2+ levels, and rapidly activating danger signal pathways to expose and release DAMPs (137–139). Consequently, modulating these factors in cancer therapy might offer new targeted treatment strategies.

Sonodynamic therapy (SDT) is a novel treatment modality that integrates low-intensity ultrasound with chemotherapeutic drugs, boasting high tissue penetration and non-invasive characteristics. Yan Zhou et al. first identified that TMZ, a standard treatment for glioma, generates significant reactive oxygen species (ROS) when exposed to ultrasound. Therefore, employing TMZ as an SDT sensitizer could enhance glioma treatment efficacy (140). Subsequent studies have shown that TMZ-based SDT causes ER expansion, mitochondrial swelling, and initiates the ER stress response (ERSR), nuclear DNA damage, and mitochondrial permeability transition pore (mPTP) opening. This process also induces “danger signals” from glioma cells, promoting the maturation and activation of bone marrow-derived dendritic cells (BMDCs), ultimately reshaping the glioma immune microenvironment and eliciting robust anti-tumor responses. Anti-tumor vaccines have recently gained attention in immunotherapy. These vaccines are created by exposing autologous dendritic cells to glioma tissue-induced ICD, wherein the dead glioma cells produced by ICDs activate astrocytoma cells and DAMPs. Upon administration to patients, the DC-presented tumor antigens trigger a specific T cell response, targeting residual astrocytoma cells, thereby reducing tumor recurrence and extending patient survival (141). Furthermore, genetically engineered virus technologies are being developed to leverage ICD, using viral vectors to deliver cytotoxic agents specifically to tumor tissues, thus inducing ICD and circumventing the blood-brain barrier’s interference with therapeutic efficacy (142).

Recently, two drugs, lurbinectedin and belantamab mafodotin, have been validated to drive ICD-mediated tumor treatment and have received FDA approval for cancer therapy (143–145). Despite the current fragmented, uncertain, and indirect nature of research on ICD-based cancer treatments, the promising potential of ICD underpins its prospective role as a pivotal component in future cancer therapies, necessitating further investigation.

## 4 Probing the synergistic impact of variegated RCD pathways for oncological treatment strategies

RCD orchestrates a range of physiological and pathological phenomena, including developmental dynamics, homeostatic integrity, and immune responses (7). It fundamentally interfaces with disease pathogenesis and healthcare outcomes. RCD plays a pivotal role in terminal cellular demise and is intricately linked to critical pathological episodes such as myocardial infarctions and neurodegenerative disorders (146–148). Furthermore, aberrations in RCD signaling cascades are implicated in uncontrolled cellular proliferation and excessive mass accumulation characteristic of neoplastic transformation (149). From an interventional standpoint, ACD is triggered by acute environmental insult, a phenomenon considered spontaneous and inevitable according to prevailing research paradigms. Conversely, the processes of RCDs are regarded as modifiable and potentially therapeutically manipulable due to their more gradual and controlled nature (150).

**Figure 4:**
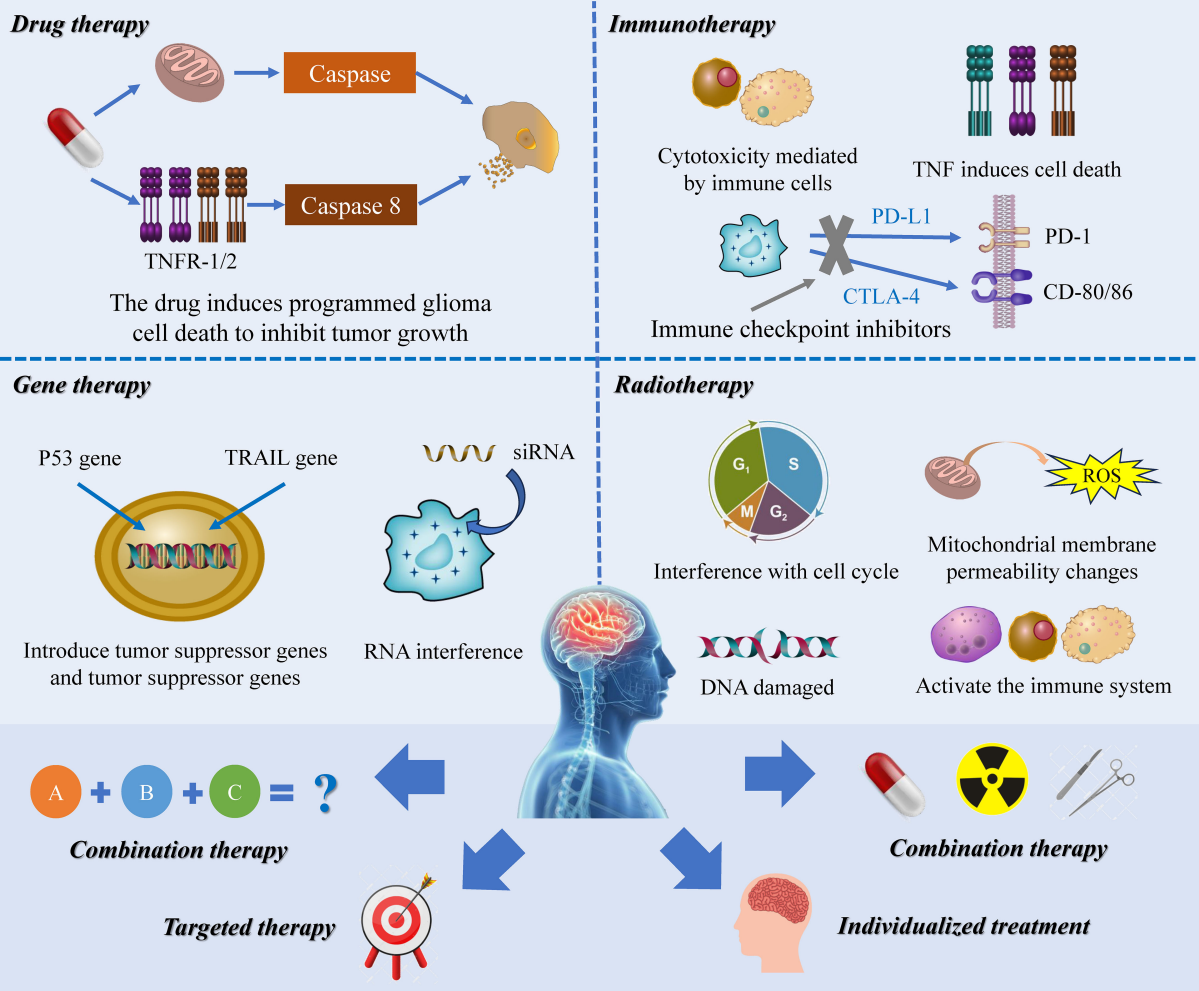
The therapeutic prospect of RCD in glioma. This image delineates the multifaceted role of RCD as a dynamic player in oncological treatments, encompassing gene therapies, precision pharmacotherapy, immune-modulatory strategies, and radiological interventions. Research highlights the intricate involvement of RCD mechanisms in mediating therapeutic efficacy. Advancing this domain, a synergistic approach that harnesses the collective force of RCD pathways and seamlessly integrates various treatment modalities holds promise as a novel paradigm in tumor management.

In recent decades, with the deepening of research on RCD, people have gradually explored the potential of various RCD pathways in treating diseases, especially tumors, using different therapeutic approaches such as targeted drug therapy, gene therapy, adjuvant RT, and immunotherapy (151, 152). Despite extensive clinical scrutiny, the translation of these insights into effective treatments has been limited due to various obstacles including challenges related to pharmacokinetics and dynamics, suboptimal trial outcomes, and safety concerns. A significant obstacle is the profound interconnectivity and complexity within mammalian RCD signaling networks; blocking one pathway pharmacologically often requires simultaneous inhibition of multiple molecular entities and cascades, thus hindering specificity. Furthermore, despite extensive research on RCD, the field remains in its early stages, with many molecular mechanisms and signaling conduits yet to be elucidated. Hasty intervention risks disrupting other physiological processes, raising doubts about the feasibility and prudence of applying RCD inhibitors in therapeutic contexts. With the emergence of one difficulty after another, researchers have gradually shifted their focus to the combination treatment and mutual tandem of RCD, which has brought new hope for tumor treatment (**Figure 4**).

RT functions primarily by inducing double-strand DNA breaks, which subsequently increase the permeability of the mitochondrial outer membrane (153). This elevation in permeability facilitates the release of pro-apoptotic proteins, thereby triggering downstream apoptotic signaling cascades. Accumulating evidence from RT research has revealed instances of anti-tumor responses in non-irradiated regions, a phenomenon known as the bystander effect. Subsequent investigations suggest that this effect may stem from the activation of anti-tumor T cells and the initiation of specific RCD mechanisms. These processes facilitate tumor eradication and, on occasion, may induce systemic bystander effects (154). Occasionally, radiation may also evoke autophagy-dependent cell death, serving as a damage control mechanism to eliminate compromised cells (155). Conversely, TMZ, an alkylating agent, inflicts DNA damage through alkyl group addition to DNA strands, culminating in cell death. The DNA damage response (DDR), particularly involving the p53 pathway, is activated during this process and leads to apoptosis. Notably, reports indicate that TMZ may also induce various forms of RCD, including necrosis, ferroptosis, and autophagy-dependent cell death (156). By leveraging these insights into radiation and TMZ-induced cell death pathways, there is potential to optimize glioblastoma treatment paradigms, thereby advancing therapeutic efficacy.

Future research endeavors may benefit from unraveling the collective dynamics among different RCD modalities. Concurrently, exploring the propagation of secondary RCD after initial cellular demise and the signal transduction of damage-associated molecular patterns (DAMPs) could provide fertile ground for pharmacological targeting.

In this immediate discourse, we focus on the interplay between autophagy and ferroptosis, particularly how autophagic mechanisms modulate the latter in response to external ferroptotic stimuli. Several studies have proposed the term “iron-dependent autophagy” to describe ferroptosis as a subset within the spectrum of autophagy. However, their distinct pathways require separate consideration; thus, this discourse will analyze their interrelation separately. Autophagy initiates ferroptosis by regulating intracellular levels of ROS, LIP, and lipid peroxides (52). Findings from these studies have primarily revolved around the xc, GPX4, and FTH systems. The phosphorylation of Beclin-1 by AMPK generates a complex with SLC7A11, thereby inhibiting its function as a Cys transporter and stimulating lipid peroxidation and iron metabolism (157). PNO1-induced autophagy enhances the synthesis and accumulation of intracellular glutamate, augmenting system Xc activity and preventing ferroptosis. Moreover, by preserving redox homeostasis, PNO1-autophagy metabolism activates system Xc responsible for cysteine synthesis and cell protection against ferroptosis (158). Owing to its interaction with distinct autophagy receptors, GPX4 has been demonstrated to be degraded following treatment with inducers of ferritin deposition or excessive copper, thereby instigating ferroptosis (159).

Within the contemporary oncological therapeutic landscape, the strategic coordination of two or more RCD pathways to combat tumor progression is increasingly evident. Lu01-m, identified as a natural compound, demonstrates a potential in stymieing prostate cancer proliferation and dissemination by propelling cells into G2/M phase arrest accompanied by DNA damage, thereby eliciting RCD pathways including necroptosis and autophagy (160). Cisplatin, a stalwart in lung cancer chemotherapy, has been documented to invoke caspase-3/GSDME-dependent pyroptosis in neoplastic cells, alongside the induction of ferroptosis through reduced glutathione levels and compromised glutathione peroxidase activity (95). Dihydroartemisinin (DHA), an artemisinin derivative, exerts its anti-tumorigenic effects by inducing cell cycle standstill and impairing angiogenesis, which culminates in the ferroptosis and apoptosis of malignant cells (161). Research by Farhan Basit and colleagues has underscored the role of BAY 87-2243, an inhibitor of mitochondrial respiratory chain complex I, in unleashing mitochondrial dysregulation, augmented ROS accumulation, lipid peroxidation, and reduced glutathione stores to foster autophagosome formation and propel ferroptotic cell death in melanoma cells (162). In the context of ovarian cancer, an investigation by Rongjun Zhang et al. unveiled the DNA-damaging capabilities of a citrus-derived substance through upregulation of poly (ADP-ribose) polymerase in a dose-dependent manner, which subsequently instigates autophagy and apoptosis. This substance also precipitates mitochondrial membrane potential diminution and ROS generation, facilitating pyroptotic cell demise (163).

The combined RCD effect has demonstrated utility in overcoming tumor resistance. Clinically, the concomitant use of resveratrol (RSV) with docetaxel (DTX) has been observed to suppress Bcl-2, amplify apoptosis, and necroptosis, thereby mitigating the drug resistance often associated with DTX monotherapy and elevating therapeutic efficacy (164). In the realm of triple-negative breast cancer, the combination of cetuximab with miR-155-5p antagonists can induce an increase in EGFR levels, promoting apoptosis and pyroptosis in breast cancer cells through the upregulation of GSDME-N and cleaved caspase-1, opening new possibilities for treatment (165).

While these insights predominantly derive from non-glioma malignancies potentially confined by the blood-brain barrier, similar therapeutic principles have been identified in glioma (**Table 3**). Yucheng Liu and colleagues have detailed an apoptotic regulatory cascade that starts with autophagy inhibition leading to mitochondrial turnover disruption, ROS amplification, DNA injury, p53 trans-activation, and culminating in apoptosis. Such suppression of autophagy propelled by TRPML1 modulation significantly hinders glioma cell proliferation. An ongoing clinical trial is investigating the synergistic impact of Haloperidol Tablets (HP) in conjunction with TMZ. TMZ has been reported to increase dopamine D2 receptor (DRD2) expression, which can cause chemotherapy resistance. As a DRD2 antagonist, Haloperidol mitigates this mechanism and enhances GBM’s susceptibility to TMZ by inducing pathways of autophagy and ferroptosis (166).

**Table 3:** List the RCD pathway inhibitors or inducers that have been approved/are undergoing clinical trials.

Targeted Drug	The main regulatory mechanism	Progress	Clinical Trial ID
Venetoclax	Inhibit BCL-2 and promote cell apoptosis.	It has been approved by the FDA and Venetoclax is primarily used for blood system malignant tumors, but its evaluation in gliomas is also underway.	/
Navitoclax	Inhibiting both BCL-2 and BCL-XL enhances cell apoptosis response.	Inhibiting both BCL-2 and BCL-XL enhances apoptotic response.	NCT03181126
Erastin	Inhibiting ferroptosis pathway	The potential therapeutic effects of ferroptosis inhibitors for GBM, which are resistant to traditional treatments, are being studied.	Not provide
Chloroquine	Inhibiting the autophagy process causes cells to accumulate toxic substances, leading to autophagy-dependent cell death.	The efficacy of chloroquine combined with TMZ and/or other therapeutic drugs for glioblastoma is being evaluated.	NCT02378532
Caspase Activators	Activate the calreticulin-dependent cytosolic pathway of necroptosis.	These drugs are designed to increase the death of tumor cells by inducing pyroptosis.	NCT02103335
Fluphenazine and TMZ Combined Therapy	Induces autophagy-dependent cell death and ferroptosis.	The DRD2 antagonist haloperidol can weaken the function of DRD2 and increase the sensitivity of GBM to TMZ by inducing lethal autophagy and ferroptosis.	NCT06218524

**Figure 5:**
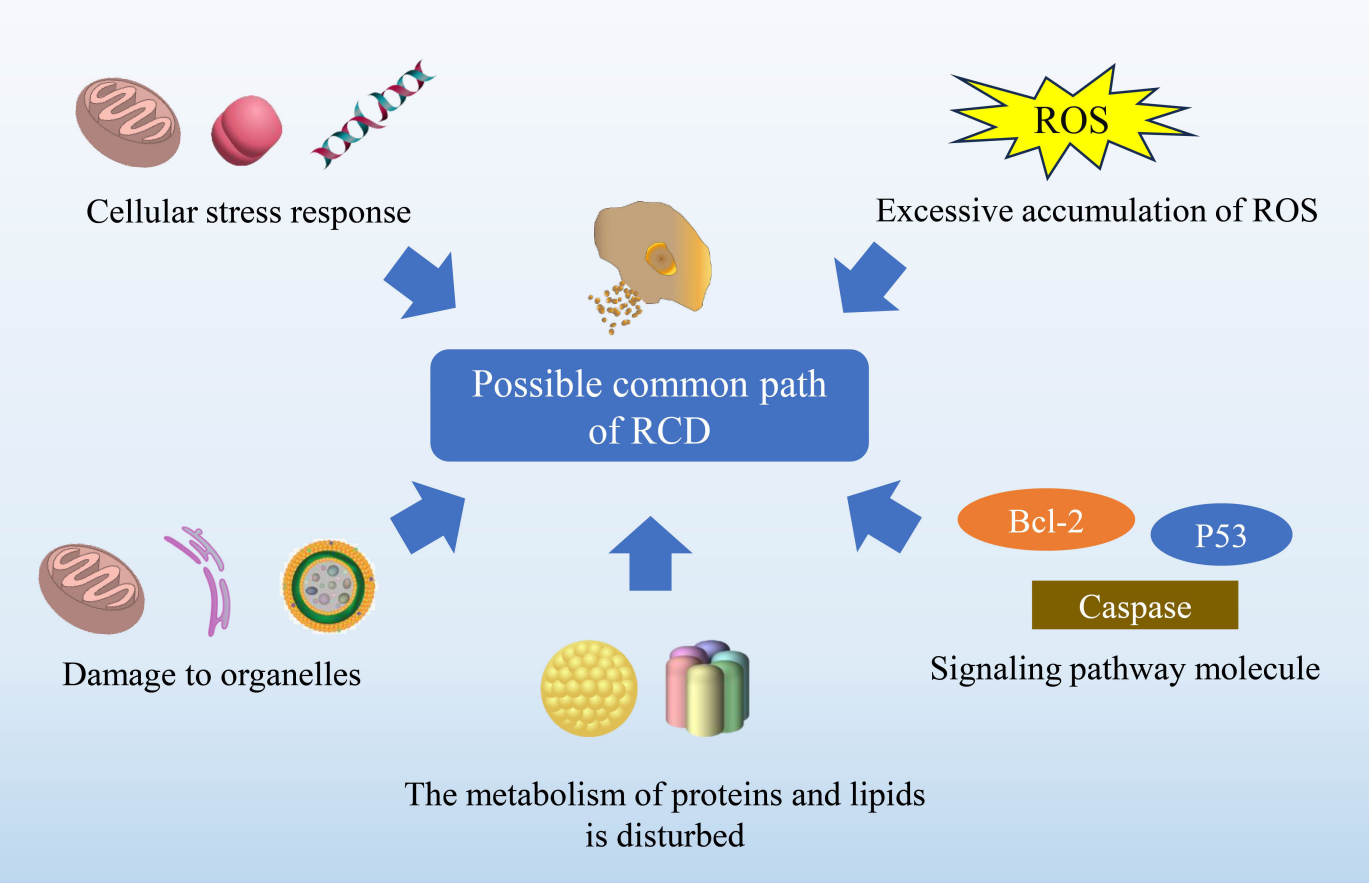
Explores potential commonalities among diverse RCD pathways. The intricate nature of RCD mechanisms remains an active area of investigation, with each pathway governed by distinct molecular signals and biological processes. As such, the full scope of autophagy-dependent cell death, ferroptosis, cuproptosis, anoikis, pyroptosis and immunogenic cell death remains to be elucidated. Nevertheless, certain fundamental characteristics might be common across these mechanisms, which include: Cellular stress response, encompassing the adaptation of proteins, lipids, DNA, and organellar structures to various forms of injury. ROS generation, where an excess can impair lipids, proteins, and DNA, hence functioning as a ubiquitous harbinger of cellular demise. Organelle dynamics involving mitochondria, lysosomes, and the endoplasmic reticulum that may engage in a coordinated response. Convergence of key molecular pathways, such as those involving tumor suppressor P53, the Bcl-2 protein family, and caspases. Perturbations in protein and lipid metabolism, which are increasingly recognized as contributors to RCD.

In summary, the mechanisms and functions of individual RCD pathways have been relatively well studied and understood, and inhibitors for these pathways have been discovered and applied (**Table 4**). However, the common mechanisms and interactions among all RCD pathways have not yet been fully elucidated. This is mainly because research in these areas is still in its early stages and is constantly evolving, encompassing a range of processes from unique molecular signals to biological processes. Despite these challenges, our discussion has revealed potential commonalities among these RCD pathways, suggesting promising directions for future research. Our hope is that these exploratory pathways will bring about key advancements in the field of tumor targeted drug therapy (**Figure 5**).

**Table 4:** A summary of small molecule inhibitors targeting key components of RCD.

Inhibitor	Process of inhibition	Illustrate
Bcl-2 family inhibitors	Apoptosis	ABT-199(Venetoclax)
Caspase inhibitor	Apoptosis	Z-VAD-FMK
RIPK1 inhibitor	Necroptosis	Necrostatin-1(Nec-1)
RIPK3 inhibitor	Necroptosis	GSK’872
Caspase-1 inhibitor	Pyroptosis	VX-765
Gasdermin D inhibitor	Pyroptosis	Under research
PI3K/mTOR inhibitor	Autophagy	Rapamycin
VPS34 inhibitor	Autophagy	3-Methyladenine(3-MA)
Iron chelating agent	Ferroptosis	Deferoxamine (DFO)
Copper chelating agent	Cuproptosis	D-Penicillamine
HSP90 inhibitor	Anoikis	NVP-AUY922
PARP inhibitor	ICD	Olaparib

## 5 Conclusion and future perspectives

Currently, in glioma investigations, there is a primary emphasis on either enhancing or impeding the autophagy cascade for autophagy-dependent cell death. While ferroptosis studies have not gained as much prominence as in other tumor types, a subset has advanced to clinical evaluation. However, research into the modalities of cuproptosis and anoikis remains relatively scarce, with the majority confined to preliminary bioinformatics analyses.

Although different forms of RCD pathways may appear distinct, there is a close connection among them. Therefore, comprehensively studying and applying multiple mechanisms could potentially become a means of targeted therapy for glioma in the future.

By regulating autophagic processes, there is potential to improve therapeutic outcomes for glioma. Current studies mainly focus on related genes, pathways, and adhesion molecules; however, there are evident limitations due to the specific nature and location of glioma. In the future, drawing upon research strategies from other tumors and proposing relevant studies on anoikis and glioma could provide valuable insights for analyzing and modulating the tumor microenvironment of gliomas. Considering the accumulation and abnormal distribution of metal ions in glioma therapy, understanding the mechanisms of ferroptosis and cuproptosis holds significant therapeutic potential. Future studies could investigate the underlying mechanisms behind the abnormal accumulation of iron and copper ions in glioma cells, which would involve developing novel therapeutic strategies targeting these metal ions to regulate apoptosis, as well as evaluating their clinical efficacy and safety.

The continuous advancement of artificial intelligence has led to the increasing utilization of deep learning in the diagnosis, grading, typing, and prognosis prediction of gliomas. Lei Jin et al. employed deep learning techniques on slide images and molecular markers stained with hematoxylin and eosin to classify gliomas, achieving an accuracy rate of 86.5% at the slide level and 87.5% at the patient level (167). In a separate investigation, Chenhua Luo et al. extracted 512 histopathological features from HE stained slides and constructed a deep learning model based on histological images to forecast glioma recurrence and patients’ survival (168). Currently, there are four commonly used methods for detecting RCD: histopathological examination, immunohistochemical staining, Terminal deoxynucleotidyl transferase dUTP Nick End Labeling (TUNEL), and Annexin V/PI staining. Among these methods, one of the most prevalent approaches is to stain and microscopically observe biopsy or surgical excision tissue specimens in order to detect changes in cell morphology (169). In the future, the utilization of machine learning and deep learning algorithms to analyze vast medical image datasets, combined with artificial intelligence’s capability to identify and extract features associated with RCD, may present a viable method for predicting glioma cell apoptosis. Integrating such a method into the diagnosis and prognosis of gliomas holds the potential to significantly improve treatment outcomes for glioma patients.

Over the years, the concept of RCD has been proposed and extensively studied. However, current research primarily focuses on analyzing and constructing clinical prediction models through bioinformatics, which has its limitations. Relying solely on large-scale data analysis and statistical methods for research may lead to data overfitting where the model becomes overly complex and fails to accurately fit new data. Furthermore, these models are constructed based on predetermined assumptions as well as patterns derived from existing data, which may potentially overlook other essential factors and underlying mechanisms. Moreover, observations and reasoning that solely rely on population statistics often disregard individual differences and heterogeneity, thereby impeding the adoption of precision therapy. Although bioinformatics tools along with predictive models offer great potential in medical research and clinical practice, it is vital to recognize their limitations. It is necessary to make efforts towards addressing these limitations while optimizing the utilization of such models. In future advancements within this field, emphasis should be placed on conducting large-scale clinical trials as well as practical applications to assess and refine both the validity and reliability of said predictive models.
